# Multifaceted role of prohibitin in cell survival and apoptosis

**DOI:** 10.1007/s10495-015-1143-z

**Published:** 2015-06-20

**Authors:** Ya-Ting Peng, Ping Chen, Ruo-Yun Ouyang, Lei Song

**Affiliations:** Department of Respiratory Medicine, Respiratory Disease Research Institute, Second XiangYa Hospital of Central South University, Changsha, 410011 People’s Republic of China; Center of Organ Transplantation, Second XiangYa Hospital of Central South University, Changsha, 410011 People’s Republic of China

**Keywords:** Prohibitin, Survival, Apoptosis

## Abstract

Human eukaryotic prohibitin (prohibitin-1 and prohibitin-2) is a membrane protein with different cellular localizations. It is involved in multiple cellular functions, including energy metabolism, proliferation, apoptosis, and senescence. The subcellular localization of prohibitin may determine its functions. Membrane prohibitin regulate the cellular signaling of membrane transport, nuclear prohibitin control transcription activation and the cell cycle, and mitochondrial prohibitin complex stabilize the mitochondrial genome and modulate mitochondrial dynamics, mitochondrial morphology, mitochondrial biogenesis, and the mitochondrial intrinsic apoptotic pathway. Moreover, prohibitin can translocates into the nucleus or the mitochondria under apoptotic signals and the subcellular shuttling of prohibitin is necessary for apoptosis process. Apoptosis is the process of programmed cell death that is important for the maintenance of normal physiological functions. Consequently, any alteration in the content, post-transcriptional modification (i.e. phosphorylation) or the nuclear or mitochondrial translocation of prohibitin may influence cell fate. Understanding the mechanisms of the expression and regulation of prohibitin may be useful for future research. This review provides an overview of the multifaceted and essential roles played by prohibitin in the regulation of cell survival and apoptosis.

## Introduction

Prohibitin, a highly conserved group of proteins, are ubiquitously expressed in many cell types and are mainly located in the mitochondria, nucleus, and the plasma membrane. Prohibitin-1 (PHB1) and prohibitin-2 (PHB2) are the two highly homologous subunits of the eukaryotic mitochondrial PHB complex. PHB1 and PHB2 are interdependent on the protein level, and loss of one simultaneously leads to the loss of the other [1, 2]. Both PHB1 and PHB2 are composed of an N-terminal transmembrane domain, an evolutionarily conserved PHB domain that is similar to that of lipid raft-associated proteins, and a C-terminal coiled-coil domain that is involved in protein–protein interactions, including the interaction between PHB1 and PHB2 as well as transcriptional regulation. At the cell plasma membrane, PHB is a transmembrane adaptor that activates downstream signal transduction [3]. In the nucleus, PHB regulates transcriptional activation and the cell cycle. At the mitochondrial inner membrane, 12–16 PHB1 and PHB2 heterodimers associate to form a ring-like macromolecular structure of approximately 1 MDa, with no homodimers detected to date. This complex is implicated in mitochondrial genome stabilization, mitochondrial morphology, oxidative stress, and apoptosis [3, 4]. Because PHB is closely associated with oxidative stress and mitochondrial dysfunction, altering the subcellular localization of PHB expression or targeting cell surface PHB may provide promising strategies for the treatment of inflammatory bowel disease, myocardium injury, diabetes, cancer and obesity [3, 5].

Apoptosis, a key regulator of tissue homeostasis, is tightly regulated by the interactions of activating and inhibitory pathways. Aberrant induction of cell apoptosis may result in neurodegenerative diseases, chronic inflammatory diseases and autoimmune diseases among others. Overexpression of PHB induces cellular resistance to various stimuli via the mitochondrial apoptotic pathway, while knockdown of PHB increases susceptibility to apoptosis stimuli. Stem cell studies also showed that ablation of PHB2 caused massive apoptosis and early embryonic lethality in mice [1, 6, 7]. However, the effect of PHB1 on cell apoptosis and survival is complicated in cases of persistent apoptosis resistance such as liver fibrosis and tumorigenesis. Notably, PHB1 is required for gonadotropin-releasing hormone (GnRH)-induced cell apoptosis of mature gonadotropins [8] and Tan IIA-induced apoptosis of activated hepatic stellate cells (HSCs) [9]. In the field of cancer, there are contradictory findings regarding the role of PHB in cancer cell survival. Some studies showed that knockdown of PHB increased cancer cell apoptosis [2, 10–15]. However, other studies found that PHB1 deficiency accelerated cancer cell growth and decreased cell apoptosis [16–19]. Intriguingly, knockdown of PHB1 increased cancer cell apoptosis in SGC7901 cells [13], but overexpression of PHB1 increased apoptosis in BGC823 cells [18], and both lines are gastric carcinoma cells. The degree of cancer cell differentiation may explain some of these differences. Overall, it seems that the expression of PHB, the stimuli and cell type may influence cell survival and apoptosis.

A series of studies suggest that subcellular localization may explain the paradoxical anti- and pro-apoptotic effect of PHB on different cell types [20]. In mouse embryonic fibroblasts (MEFs) and human cervical cancer HeLa cells, PHB1 and PHB2 are mainly localized in the mitochondria, and complete silencing of PHB1 or PHB2 causes mitochondrial network fragmentation followed by increased mitochondria-mediated cell apoptosis under intrinsic and extrinsic apoptotic stimuli [1, 2, 21]. In paclitaxel-resistant lung cancer cells, PHB1 is mainly localized in the mitochondria and the plasma membrane, and knockdown of PHB1 activates the intrinsic apoptotic pathway following paclitaxel treatment both in vitro and in vivo [22]. Additionally, PHB2 is mainly localized in the mitochondria of pluripotent mouse embryonic stem (ES) cells, and knockdown of PHB2 causes induction of apoptosis in mouse ES and human pluripotent stem (iPS) cells. Ectopic expression of PHB2, but not a mitochondria-targeted signal-mutated version of PHB2, effectively suppresses apoptosis, suggesting that PHB2 localized in the mitochondria is crucial to the survival of pluripotent ES cells [23]. However, in invasive and noninvasive breast cancer cells, PHB1 is mainly confined to the nucleus, in contrast to normal epithelial breast cells in which PHB1 is located primarily in the mitochondria [24]. In the MCF-7 and T47D breast cancer cell lines, PHB1 is mainly located in the nucleus [25, 26]; in the LNCaP prostate cancer cell line and the MG-63 osteosarcoma cell line, the presence of PHB1 staining in the nucleus was also noted [27, 28]. Silencing PHB1 enhanced the percentage of proliferating cells in the population, as reflected by increased Brd/Urd incorporation, increased cell distribution in S phase and decreased cell distribution in the G1–G0 phase of the cell cycle in MCF-7 cells [26]. Consistently, in the HCT 116 human colon carcinoma cell line, PHB1 was exclusively expressed in the nucleus, while PHB2 appeared to be present in both the cytoplasm and the nucleus, and knockdown of PHB1 or PHB2 inhibited apoptosis induced by the topoisomerase I inhibitor camptothecin in this cell type [29]. In a recent study of human bladder cancer (BC), PHB1 was identified as an important regulator during BC tumorigenesis in that it is frequently overexpressed in BC tissues and significantly correlated with poor prognosis in BC patients. Interestingly, the overexpressed PHB1 was primarily found within the mitochondria. It was also found that Akt phosphorylates PHB1 Thr^258^ in the cytoplasm without influencing the total PHB 1protein level and promotes PHB1 mitochondrial translocation to induce BC proliferation [30]. All of these observations suggest that in most cell types, mitochondrial and membrane-associated PHB is associated with an anti-apoptosis function or tumorigenesis. Nevertheless, nuclear PHB displays pro-apoptosis or anti-tumorigenic properties. The subcellular localization and the regulation of the transcription, translation, and posttranslational modification of PHB may determine the final effect of PHB on cell survival and apoptosis (Table 1).

**Table 1:** PHB-mediated anti- and pro-apoptotic activity in vivo and in vitro studies

Modification of members		Cell type/model	Apoptosis	Effect on mitochondria (mt)	Overall impact	Reference
*Non-cancer*						
Knockdown	PHB1	Mice (gonadotrope-specific depletion)	**↓**	NO	Reproductive ability↓	[8]
	PHB1	HSC-T6 cells (Tan IIA)	**↓**	NO	NO	[9]
Over-expression	PHB	Mice (cardiac-specific overexpression + MI injury)	**↓**	mt fission ↓	Myocardial infarction size↓Cardiac function↑	[66, 67]
	PHB1	Rats (Spinal cord injury)	**↓**	Respiration rates↑ electron transport↑ cleaved 3↓ Bax/Bcl-2↓ ATP↑	Locomotor function↑ ER stress↓ PI3K/AKT, ERK↑ NF-κB↓	[57]
	PHB1	Mice (forebrain ischemia)	**↓**	Cytochrome c release↓ caspase-3↓	Hippocampal function↑	[107]
	PHB1	Neuronal (rotenone glutamate)	**↓**	RC↑	Hippocampal damage↓	[96]
	PHB1	Rats (UUO)	**↓**	NO	ECM↓ Renal tubulointerstitial fibrosis↓	[108]
	PHB1	HUVEC (glyLDL)	**↓**	Cytochrome c release↓ caspase-3 Bax/Bcl-2↓	AKT↓ p-AKT↓	[58]
	PHB1	Pancreatic β cells (ethanol)	**↓**	Cleaved caspase-3↓	NO	[123]
	PHB1	Cardiomyocytes (hypoxia)	**↓**	Cytochrome c release↓ Bax/Bcl-2↓ MMP↑	Promote cell survival	[62]
	PHB1	Cardiomyocytes (H_2_O_2_)	**↓**	Cytochrome c release↓ MMP↑ ATP↑	NO	[63]
	PHB1	Undifferentiated GCs (STS)	**↓**	Caspase-3, Bax, Bak↓ Bcl-2, Bcl-xl↑ mt content↑Cleaved caspase-3↓	ERK↑	[59, 124]
	PHB1	Undifferentiated GCs (ceramide)	**↓**	Cytochrome c release↓ Cleaved caspase-3↓MMP↑	NO	[60, 61]
Knockdown	PHB1	Mice (injurious stimuli)	**↑**	Caspase-3↑ MMP↓	NO	[96]
	PHB2	Mice (hippocampus-specific depletion)	**↑**	Aberrant cristae morphology, RC↓ mt DNA copy number↓	Behavioral, cognitive impairments↑ GSK3↓ ERK, JNK, AKT↑ Neurodegeneration↑	[68]
	PHB2	Mice(EpSCs-specific depletion)	**↑**	NO	Die fast	[6]
	PHB1	Mice (liver-specific depletion)	**↑**	Aberrant cristae morphology, caspase-3↑ lipid peroxidation↑	Hepatocyte proliferation↑ fibrosis↑ Liver progenitor cell expansion↑ HCC↑	[15]
	PHB1	ARPE-19 cells	**↑**	Caspase-9↑ AIF, BAK↑ Bcl-xl↓ fragmented mt↑	NO	[43]
	PHB2	MEFs (intrinsic and extrinsic apoptotic stimuli)	**↑**	Aberrant cristae morphology, caspase-3↑ L-Opa1↓ no influence on MMP, RC	Stop growing, cellular proliferation↓	[1]
	PHB2	Mouse embryonic stem cells and human iPS cells	**↑**	Aberrant cristae morphology, impaired mt dynamics, ATP↓ MMP↓ ROS↓	NO	[23]
	PHB1	HUVEC (glyLDL)	**↑**	Cytochrome c release↑ Caspase-3↑ Bax/Bcl-2↑	AKT↑	[58]
	PHB2	PC12 cells (H_2_O_2_)	**↑**	NO	NO	[125]
	PHB1	BAECs	**↑**	MMP↓ RC↓	Senescence↑ Angiogenic ability↓ AKT↑	[95]
	PHB2	β-cells	**↑**	RC, mtDNA copy number↓ L-Opa1↓ fragmented mt↑	Insulin supply↓	[69]
	PHB	Kit225 (cytokine deprivation)	**↑**	MMP↓	NO	[115]
	PHB1	Undifferentiated GCs (STS)	**↑**	Aberrant cristae morphology Cleaved caspase-3↑	NO	[59, 124]
	PHB1	Undifferentiated GCs (ceramide)	**↑**	Cytochrome c release↑ Cleaved caspase-3↑	NO	[60]
*Cancer*						
Over-expression	PHB1	Gastric cancer cells (BGC823)	**↑**	Caspase-3, caspase-9↑ Bax↑ Bcl-2↓	G1↓ G2,S phase↑ cellular proliferation↓	[18]
Knockdown	PHB1	Gastric cancer cells (SGC7901)	**↑**	NO	G1/S phase↑ cellular proliferation↓	[13]
	PHB1	Mice (xenograft gastric cancer)	**↑**	Caspase-3,9↑ Bax↑ Bcl-2↓	Transplanted tumor growth↓	[13]
	PHB1	A549TR (paclitaxel)	**↑**	Caspase-3/7↑ caspase-9↑ Bcl-2↓ cleaved PARP↑	Rescue paclitaxel sensitivity	[22]
	PHB1	Mice (xenograft A549TR + paclitaxel)	**↑**	Caspase-3/7↑	MDR activity↑ tumor volumes↓	[22]
	PHB1	Ovarian cancer cells (STS)	**↑**	Aberrant cristae morphology	NO	[10]
	PHB1	HaCaT (UVB)	**↑**	NO	NO	[11]
	PHB1	Hepatoma cells	**↑**	NO	Cellular proliferation↓	[14]
	PHB1	Murine non-transformed AML12 cells	**↑**	NO	Cellular proliferation↑ cyclin D1↑	[15]
	PHB1	HaCaT (anthralin)	**↑**	MMP↓	Sub-G1 phase↑G1-phase↓	[12]
	PHB2	Hela cells	**↑**	Cytochrome c release↑ Fragmented mt↑ MMP↓	NO	[2]
	PHB1	Hela cells	**↑**	mtDNA disorganization, copy number↓	NO	[84]
	PHB1	Hela cells (aurilide)	**↑**	NO	NO	[21]
	PHB1	Human pancreatic cancer cells	**↓**	NO	NO	[17]
	PHB1	NB4-R1 cells	**↓**	NO	NO	[19]
	PHB1	Human colon carcinoma cells	**↓**	NO	NO	[29]
	PHB1	Human Jurkat T leukemia cells	**↓**	NO	NO	[16]

Abbreviations: *A549TR* paclitaxel-resistant lung cancer cell, *ARPE-19* human retinal pigment epithelial cells, *BAECs* bovine aortic endothelial cells, *ECM* extra-cellular matrix, *ER* endoplasmic reticulum, *EpSCs* epidermal progenitor/stem cells, *GCs* granulosa cells, *glyLDL* glycated low-density lipoproteins, *HCC* hepatocellular carcinoma, *HUVEC* human umbilical vein endothelial cells, *HaCaT* human keratinocytes cells, *Kit225* IL-2-dependent human T cell line, *MDR* multidrug resistance, *MI* myocardial ischemia, *MMP* mitochondrial membrane potential, *MEFs* mouse embryonic fibroblasts, *NB4-R1* cells retinoic acid-resistant acute promyelocytic leukemia cell line, *NO* not observed, *PC12 cell* neural cell line, *PARP* poly ADP-ribose polymerase, *RC* respiratory complex, *STS* staurosporine, *Tan IIA* tanshinone IIA, *UUO* unilateral ureteral obstruction

## PHB and control of apoptosis in the membrane

At the inner cell membrane, PHB1 can mediate the cellular signaling of membrane transport. In cancer cells, PHB is required for plasma membrane association of C-Raf and is indispensable for the activation of the Ras-mediated Raf-MEK-ERK signaling pathway, which may modulate cancer cell survival and migration [31–33]. Rocaglamide (a flavagline), a novel anticancer agent, can sensitize extrinsically induced apoptosis in resistant cancer cells [34]. Recently, rocaglamide was found to selectively bind to PHB1 and PHB2 with nanomolar affinity and to influence the membrane localization of PHB1 and PHB2 in the human T cell leukemic cell line Jurkat, the human cervical cancer cell line HeLa and the pancreatic cancer cell line AsPC-1. This binding further disrupts the PHB/C-Raf interaction at the inner cell membrane, arrests the cell cycle at the G0/G1 phase and results in inactivation of the oncogenic Raf-MEK-ERK signaling pathway [32, 35, 36]. Although rocaglamide-induced loss of PHB1 and PHB2 membrane localization did not cause mitochondrial fragmentation in Jurkat or and HeLa cells and did not induce distinct apoptosis in these cells, rocaglamide treatment downregulated the G1/S regulatory proteins cyclin D3, CDK4, CDK6, and cdc25A and inhibited cell cycle progression at the G1/S phase [35]. However, studies have identified membrane-associated PHB1 as a pro-apoptosis mediator in liver fibrosis. Appropriate removal of HSCs by apoptosis is a potential strategy for the treatment of liver fibrosis. In HSCs, upon Tan IIA stimulation, there is increased translocation of C-Raf protein from the cytoplasm to the membrane as well as predominant PHB1 localization in the membrane. Knockdown of PHB1 causes loss of PHB1 membrane localization and disruption of the PHB1/C-Raf complex, which further attenuate Tan IIA-induced apoptosis of activated HSCs [9].

**Fig. 1:**
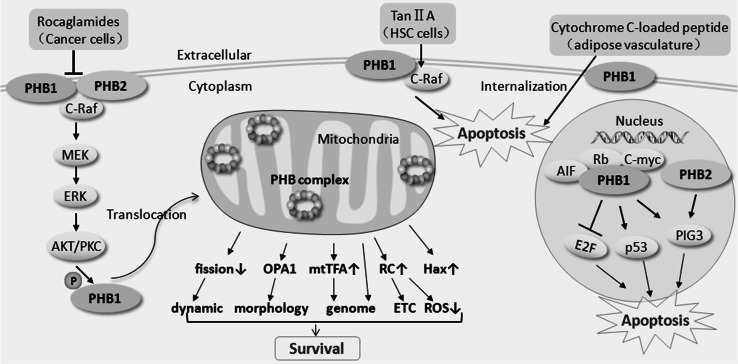
Schematic demonstration of PHB-mediated survival or apoptosis responses in multiple cellular compartments of different cell types and molecular mechanism involved. At the outer plasma membrane, PHB1 mediates the endocytosis of a pro-apoptotic peptide in the induction of apoptosis in the adipose vasculature. At the inner plasma membrane, membrane-localized PHB1 or PHB2 associate with C-Raf and is involved in the activation of the Raf-mitogen-associated protein kinase kinase (MEK)-extracellular signal-regulated kinase (ERK) signaling pathway, which can promote cancer cell survival. The serine/threonine-protein kinase (AKT) can phosphorylate PHB1 in the cytoplasm and promotes PHB1 mitochondrial translocation to induce bladder cancer proliferation. The anticancer agent rocaglamides mediates the pro-apoptotic action by disrupting the PHB1/C-Raf or PHB2/C-Raf interaction. However, increased membrane localization of PHB1 and the PHB1/C-Raf complex in activated hepatic stellate cells may promote Tan IIA-induced apoptosis. The ring-shaped PHB complex in mitochondria may regulate mitochondrial dynamics, mitochondrial morphology, mitochondrial genome, the electron transport chain (ETC), reactive oxygen species (ROS) homeostasis, and anti-apoptotic proteins, which further prevent mitochondria-mediated apoptosis. Nuclear PHB1 co-localizes with many transcription factors, such as p53, Rb, E2F, AIF, c-myc, and c-fos, which may influence the cell cycle and tumor growth in a coordinated manner. PHB1 can upregulate p53-mediated transcription but repress E2F activity in B cell lymphoma cells, and overexpression of PHB1 protects cells from camptothecin-induced apoptosis. In the absence of p53, both PHB1 and PHB2 can contribute to P53 inducible gene 3 (PIG3)-mediated apoptosis by increasing the transcription of PIG3

On the cell surface, PHB1 also acts as specific receptor in white fat vessels. PHB1 is abundant in the endothelial cells of white adipose tissue in mice and humans, and the membrane-associated PHB1 receptor is the target of an adipose-specific peptide [37]. Thus, PHB1 can mediate the internalization of the adipose-specific peptide [38]. A subsequent study showed that cytochrome C-loaded PHB1-targeted nanoparticles (PTNPs) induce apoptosis in the endothelial cells of white fat vessels in vivo and prevent diet-induced obesity (DIO) in a dose-dependent manner in vitro. In addition, the delivery of a pro-apoptotic peptide through PTNPs had no detectable liver toxicity and only induced apoptosis in the adipose vasculature, not in endothelial cells in other organs (such as heart, lung, liver, spleen, and kidney), suggesting that a specific PHB1 receptor complex in the white adipose vasculature may contribute to the induction of apoptosis as a mechanism of treatment for obesity [38, 39]. The membrane localization of PHB1 has an unusual ability to control apoptosis; it clearly not only functions as a transmembrane signal receptor in the survival of cancer cells and the apoptosis of HSCs but also mediates endocytosis in the induction of apoptosis in the adipose vasculature (Fig. 1).

## PHB and its transcriptional regulation in the nucleus

Multiple lines of evidence indicate that nuclear-localized PHB protein is a unique regulator of specific transcription factors and cell cycle-associated proteins. This transcriptional regulation function of PHB1 in the nucleus may provide a link between the proliferative and apoptotic pathways [25, 40, 41]. In general, PHB1 co-localized with p53, Rb, E2F, AIF, c-myc, and c-fos in the nucleus [25, 27, 42–45]. These tumor-suppressor and oncogene proteins are of great importance for mammalian cell apoptosis and survival, and they usually function in a coordinated manner [46]. In MCF7 cells, overexpression of PHB1 increased p53-mediated and repressed E2F1-mediated transcriptional activity [25]. In B cell lymphoma Ramos cells, overexpression of PHB1 upregulated p53-mediated transcription and repressed E2F activity. It also protects cells from camptothecin-induced apoptosis [47]. PHB1 does not affect the receptor-mediated death of Ramos cells through either the Fas or TNF pathways [47]. It is intriguing that the PHB1 coiled–coil domain between amino acids 177 and 217 can mediate its homo-dimerization or its interaction with heterologous proteins such as E2F1 and HDAC1, and it can also repress E2F1-mediated transcription [48]. However, a putative PHB1 coiled-coil domain induced caspase-dependent apoptosis of MCF7 and WI-38 cells, although overexpression of full-length PHB1 protein induced growth arrest, not apoptosis [48]. In addition, in the human colon carcinoma cell line HCT 116, PHB1 and PHB2 contribute to PIG3-mediated apoptosis by binding to the P53 inducible gene 3 (PIG3) promoter (TGYCC)15 motif and initiating the transcription of PIG3 in the absence of p53 [29]. Therefore, PHB is involved in both p53-dependent and independent apoptotic processes (Fig. 1).

## PHB and nuclear trafficking and apoptosis

PHB is a shuttle protein that shuttles between subcellular compartments [43]. Newly synthesized PHB1 and PHB2 in the cytoplasm or mitochondrially located PHB1 and PHB2 need to pass through the nuclear pore complex to translocate into the nucleus, where they act as a transcriptional modulator. It has been demonstrated that human PHB2 contains both an uncleavable mitochondrial targeting sequence (MTS) at its N terminus and a nuclear localization sequence at its C terminus [2]. Although human PHB1 does not possess the MTS, the N-terminal portion of PHB1 is also the sole determinant of mitochondrial targeting [2]. Moreover, the coiled-coil structure of the alpha helices in the C terminus contains a leucine/isoleucine-rich nuclear export sequence (NES) that facilitates the export of PHB to the mitochondria or cytoplasm [5, 49]. This specific structure of PHB proteins allows active shuttling between the organelles.

**Fig. 2:**
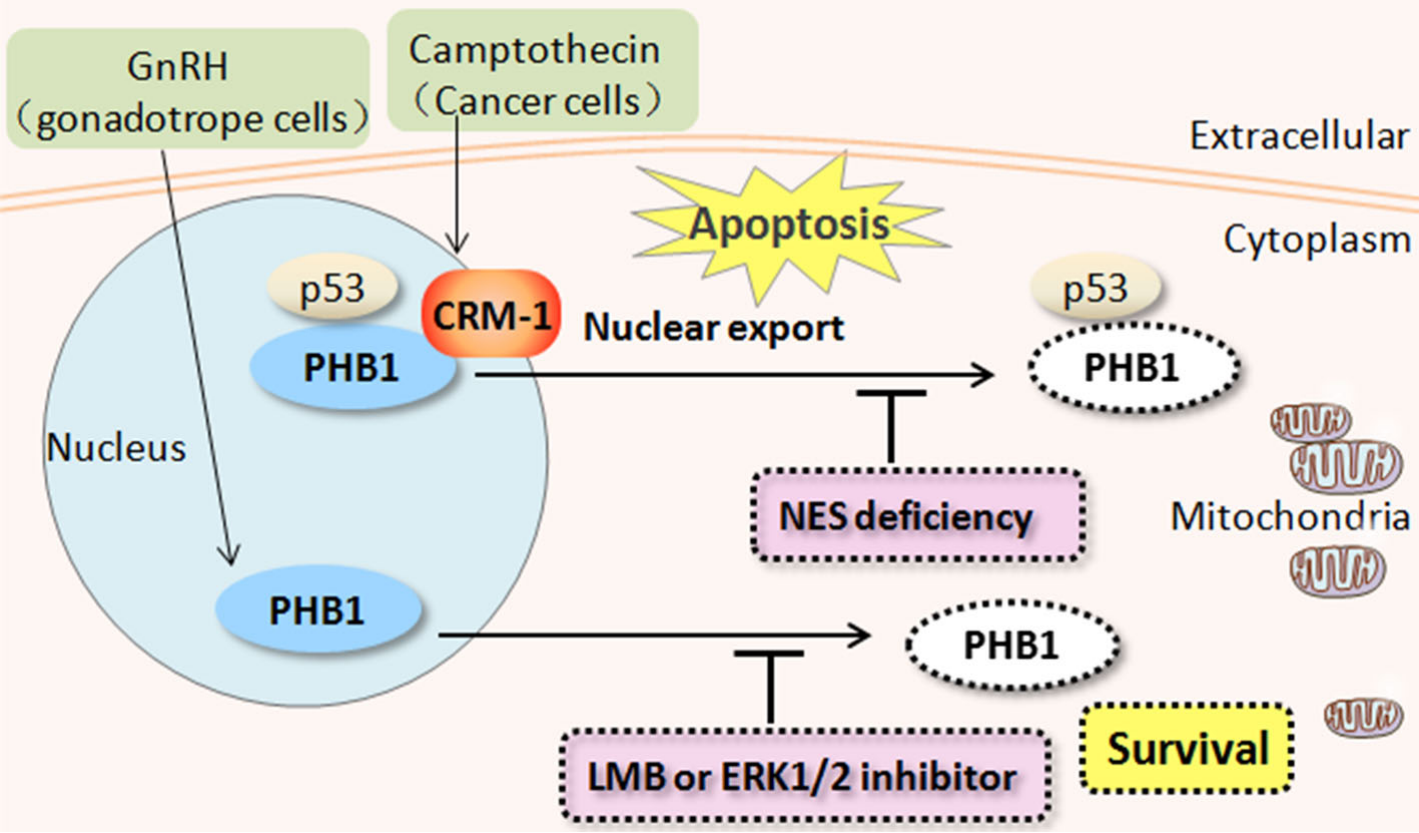
The effect of inhibition of nuclear export of PHB1 on cell apoptosis. In camptothecin-induced apoptosis of human breast carcinoma cells, both PHB1 and p53 undergo export from the nucleus to the cytoplasm upon receiving apoptotic signals. Nuclear export is dependent on the exportin 1 (CRM1). Inhibition of the nuclear export of PHB1 by delivery of a PHB1- nuclear export sequence (NES) peptide lacking the NES prevents camptothecin-mediated apoptosis and it didn’t affect the translocation of p53. In gonadotropin-releasing hormone (GnRH)-induced apoptosis of mature gonadotrope cells, leptomycin (LMB) or the extracellular signal–regulated kinase 1/2 (ERK1/2) inhibitor U0126 inhibit the nuclear export of PHB1 and decrease apoptosis

**Fig. 3:**
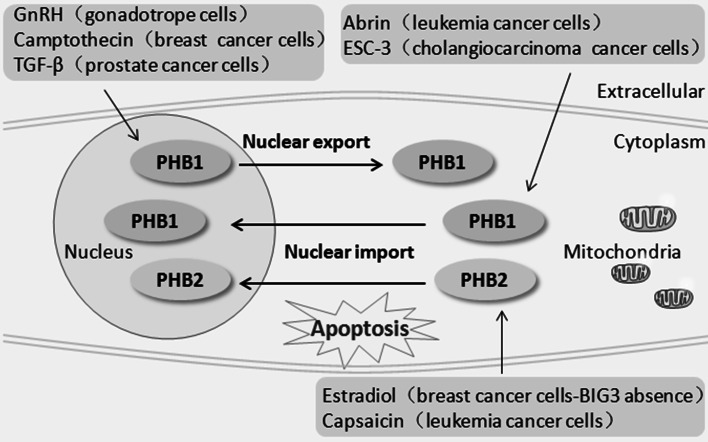
The subcellular shuttling of PHB during cell apoptosis. During the apoptosis of gonadotrope cells induced by GnRH and apoptosis of cancer cells induced by camptothecin or TGF-β, PHB1 is exported from the nucleus to the cytoplasm or mitochondria. However, during the apoptosis of cancer cells induced by abrin or ESC-3, PHB1 translocates from the cytoplasm or mitochondria to the nucleus. During the apoptosis of cancer cells induced by estradiol or capsaicin, PHB2 translocates from the cytoplasm or mitochondria to the nucleus

The subcellular localization of PHB is affected by apoptotic signals, and inhibition of this translocation movement affects apoptosis. Proteomic analysis of apoptosis induced by the nuclear export inhibitor leptomycin (LMB) of HeLa cells showed increased nuclear sequestration of PHB1 and Hsp27 and significant cytoplasmic overexpression of PHB1 did not prevent LMB-induced apoptosis, but overexpression of Hsp27 partly inhibited LMB-induced apoptosis [50]. The nuclear export of PHB1 is accompanied by many apoptotic events. In androgen-sensitive and TGF-β-responsive human prostate cancer cells, TGF-β treatment promoted PHB1 export from the nucleus to the cytosol, which preceded apoptotic cell death [51]. In camptothecin-induced apoptosis of the human breast carcinoma cell line T47D, both PHB1 and p53 undergo export from the nucleus to the cytoplasm after receiving apoptotic signals, and the nuclear exit of PHB1 and p53 correlate with the onset of apoptosis, as marked by PARP cleavage [25]. A later study from the same group showed that in the human osteosarcoma cell line Saos-2, the breast cancer cell line MDA-MB231, and the non-small cell lung carcinoma cell line H1299, camptothecin-induced nuclear export of PHB1 does not require p53 [49]. However, the export of PHB1 from the nucleus under camptothecin treatment in the breast cancer cell lines MCF-7 and T47D and the human fetal lung fibroblast cell line WI-38 is dependent on the protein exportin 1 (CRM1) [49], a member of the importin family. Furthermore, in MCF-7 cells, inhibition of the nuclear export of PHB1 by delivery of a PHB-NES peptide without the NES prevents camptothecin-mediated apoptosis [49]. In mature gonadotropes, apoptosis of gonadotrope cells is crucial for the normal development and function of the reproductive axis. GnRH upregulates the expression of PHB1 mRNA via the JNK pathway and induces PHB1 nuclear export through ERK activation. The activation of the JNK pathway and the subsequent activation of the pro-apoptotic factors BAX and HRK, as well as the nuclear export of PHB1, is indispensable for the GnRH-induced apoptosis of murine gonadotrope-derived LβT2 cell lines; LMB or the ERK1/2 inhibitor U0126 inhibited the nuclear export of PHB1 and reduced the levels of cleaved PARP under GnRH treatment [8] (Fig. 2). In androgen-independent prostate cancer cells, estrogen activates ERα and promotes mitochondrial-nuclear PHB1 translocation, leading to cancer cell resistance to paclitaxel [52]. However, the translocation of PHB1 from the cytoplasm to the nucleus is also closely related to other apoptotic events. In abrin (ABR)-induced apoptosis of human Jurkat T leukemia cancer cells, downregulation of PHB1 delays ABR-triggered cell apoptosis [16]. In the early stages of apoptosis, PHB1 is upregulated by ABR through the JNK/SAPK signaling pathway, which turns on the expression of the pro-apoptotic gene Bax via the accumulation and translocation of the PHB1-p53 complex to the nucleus and the enhanced transcriptional activity of p53 on Bax [16]. During apoptosis induced by ESC-3, an active ingredient of crocodile bile, in human cholangiocarcinoma Mz-ChA-1 cells, PHB1 is dramatically downregulated, and the region of co-localization between PHB1 and AIF, p53, Rb, and c-fos shifted from the cytoplasm to the nucleus. In this case, PHB1 was expressed primarily in the cytoplasm and only slightly in the nucleus of Mz-ChA-1 cells [45]. In human breast cancer cells, the absence of brefeldin A-inhibited guanine nucleotide-exchange protein 3 (BIG3) promoted the translocation of PHB2 to the nucleus after estradiol stimulation and resulted in the suppression of cancer cell growth. In contrast, PHB2 was trapped in the cytoplasm in the presence of BIG3 with or without estradiol stimulation, resulting in the constitutive activation of ER signaling and tumorigenesis [53]. Capsaicin, a potential anticancer agent, can directly bind to PHB2 and induced the dissociation of PHB2 from adenine nucleotide translocator 2 (ANT2), thereby promoting the nuclear translocation of PHB2 and cancer cell apoptosis [54] (Fig. 3). Moreover, during the differentiation of human neuroblastoma SK-N-SH cells, the region of colocalization of PHB1 with p53, Rb, c-myc, and c-fos shifted from the nucleolus to the cytoplasm [55]. In summary, the nuclear shuttling movement of PHB correlates with signal transduction, cellular proliferation, apoptosis, and differentiation.

## PHB and its anti-apoptotic role in mitochondria

Mitochondria are essential organelles for all eukaryotic cells. They execute the intrinsic apoptosis pathway in response to various types of cell damage [56]. In multiple studies, overexpression of PHB1 markedly prevented mitochondria-mediated apoptosis triggered by apoptotic stimuli. The main mechanism of this protective role is PHB1-mediated inhibition of the intrinsic apoptotic pathway by maintaining the mitochondrial transmembrane potential, inhibiting cytochrome c release and caspase-3 activation, and enhancing the transcription and translation of anti-apoptotic genes such as Bcl-2 and Bcl-xl [57–63]. Located in the mitochondrial inner membrane, the PHB complex maintains mitochondrial dynamics and morphology [64], which are essential for mitochondrial function and cell survival [65]. In cardiomyocytes, forced expression of PHB1 attenuated mitochondrial fission and apoptosis induced by H_2_O_2_, and cardiac-specific PHB1 transgenic mice showed reduced mitochondrial fission and myocardial infarction sizes after myocardial infarction injury [66]. Forced expression of PHB2 also attenuated mitochondrial fission and apoptosis of cardiomyocytes induced by anoxic conditions in vivo and vitro [67]. Ultrastructural studies have observed accumulated vesicular structures, striking absence of lamellar-shaped cristae, impaired mitochondrial dynamics and augmented apoptosis in PHB2-deficient cells [1, 23, 68, 69]. Knockdown of PHB2 also induced loss of the longer Opa1 isoforms [1, 23, 69]. Anchored on the inner membrane of mitochondria, Opa1 is required for the mitochondrial fusion and cristae integrity, and loss of Opa1 commits cells to apoptosis without any other stimuli [70, 71]. The cellular defects observed in the PHB2-deficient cells are attributed to the accelerated proteolytic cleavage of Opa1 L-isoforms to S-isoforms (two long isoforms, L-Opa1; and three short isoforms, S-Opa1) because ectopic expression of a proteolysis-resistant Opa1 mutant restored mitochondrial function and prevented apoptosis [1, 21]. It is clear that a pre-existing mitochondrial morphology deficiency may render cells more susceptible to apoptotic stimulation under PHB2 depletion [1].

In the early stages of apoptosis, mitochondrial cristae are restructured, facilitating cytochrome c release from the crystal lumen to the peripheral intermembrane space (IMS) [72, 73]. Importantly, Opa1 self-assembly controls the “open” and “closed” states of the crista junction. The oligomerization of L-Opa1 and S-Opa1 along with wider crista junctions can block cytochrome c release and apoptosis [74, 75]. Another key step in initiating apoptosis is the dissociation of the cytochrome c/cardiolipin complex and the complete release of free cytochrome c into the cytosol [76, 77], a process that can also be controlled by the lipid-scaffold function of the PHB complex [4]. Indeed, as a structuring scaffold, the PHB complex may govern spatial membrane organization and regulate membrane protein activities, both of which are required for optimal mitochondrial morphology and function [1, 4, 78]. Moreover, the protection against apoptosis by Opa1 is independent of mitochondrial fusion [74], and neither decreased membrane potential nor respiratory activity was observed in the absence of PHB2, suggesting that the Opa1-dependent branch of apoptosis is involved in the activation of intrinsic apoptotic pathway [1].

An intriguing study in toxicology may provide further information about the relationship between PHB complex and Opa1 [21, 79]. Aurilide, a potent cytotoxic natural marine product, inhibits the function of PHB complex and accelerates the proteolytic processing of Opa1, results in mitochondrial fragmentation and apoptotic cell death in Hela cells [21]. However, aurilide selectively and directly binds to PHB1 in the mitochondria and selectively disrupts the PHB-m-AAA protease complex interaction rather than the PHB1-PHB2 interaction [21]. It has been demonstrated that the human m-AAA protease has limited effects on Opa1 processing in human cells [21, 80–82], which is in contrast to the effect in yeast [83]. The mitochondrial PHB complex may indirectly regulate the proteolytic processing or localization of Opa1 [21, 84].

The mitochondrial genome contains 37 genes encoding 22 mitochondrial tRNAs, 2 mitochondrial rRNAs and 13 proteins involved in the electron transport chain (ETC) that are present in complexes I, III, IV and V (not II). The mitochondrial genome is important for the synthesis of the respiratory complex and maintenance of normal mitochondrial function [85]. A comprehensive DNA repair system and sufficient mitochondrial biogenesis facilitate cell survival in the presence of apoptotic stimuli [86, 87]. In contrast to nuclear DNA, mitochondrial DNA (mtDNA) lacks introns and protective histones and is vulnerable to oxidative damage due to its close proximity to the inner membrane where reactive oxygen species (ROS) are continually produced. The major protein component of mtDNA nucleoids, mitochondrial transcription factor A (mtTFA), is involved in the repair and maintenance of mtDNA and mitochondrial biogenesis [88–90]. MtTFA knockout animals showed decreased mtDNA expression, which leads to severe respiratory chain deficiency as well as massive induction of apoptosis [91]. Importantly, mitochondrial nucleoids are also located in the inner mitochondrial membrane (IMM), and the PHB complex is a peripheral component of mitochondrial nucleoids [92]. Therefore, the PHB complex may affect the attachment of nucleoids to the IMM through functional partitioning. Studies showed that depletion of PHB1 or PHB2 causes a reduction in the mtDNA copy number and predisposes cells to apoptosis [68, 69, 84]. Moreover, the PHB complex maintains the copy number of mtDNA through mtTFA regulation [84], but the PHB complex may affect the organization of mitochondrial nucleoids neither by mtTFA-dependent nor by Opa1-dependent pathway [84]. Interestingly, PHB1 may preferentially maintain the nucleoid organization compared with PHB2 [84]. Taken together, these data show that the PHB complex maintains the organization and stability of mitochondrial genome and functions as essential survival factors.

As discussed above, the disorganization of mitochondrial nucleoids by PHB1 or PHB2 depletion leads to disturbed mitochondrial biogenesis. When PHB1 expression was decreased by siRNA transfection, the expression levels of mtDNA-encoded subunits I and II (CoxI, CoxII) and nDNA-encoded subunits IV and VIb (CoxIV, CoxVIb), as well as Cox activity, all decreased [93]. Studies also confirmed that PHB1 or PHB2 silencing suppresses respiratory chain complex activity [68, 69, 94] and partially blocks mitochondrial electron transport [95], whereas overexpression of PHB1 increased complex I and complex IV activity [96], improved respiration rates and restored ATP formation [57, 63]. Interestingly, PHB1 is found to physically interact with respiratory chain complex I as well as cytochrome c oxidase subunit II (CoxII) in the IMM [93, 97], which means that the PHB complex is required for the detailed assembly and stability of respiratory chain components [64, 98], either by affecting mitochondrial biogenesis or in a chaperon-like manner. Recent research revealed that the respiratory chain can modulate apoptosis in a context-dependent manner with no impact on ATP production [99]. Respiratory chain dysfunction caused by mtDNA mutations enhanced ROS production and changed the complement of anti-apoptotic proteins in the mitochondria [99]. Therefore, in PHB-deficient cells, blockage of the ETC caused by partial damage to respiratory chain complexes strongly enhanced the sensitization of these cells to the induction of apoptosis.

Mitochondria are a major source of ROS, and the PHB complex is involved in the regulation of ROS. ROS are a byproduct of mitochondrial respiration, and their formation is mainly due to natural electron leakage occurring at respiratory chain complexes I, II and III. Continued oxidative stress can lead to mtDNA, lipid and protein damage and defects in signaling transduction pathways, affecting the onset of apoptotic cell death [100]. Multiple studies have documented that PHB1 expression is enhanced in various stress conditions [62, 93, 96, 101, 102] and is decreased in intestinal epithelial cells during oxidative stress [103]. During oxidative stress, PHB1 can be exported from the nucleus to the mitochondria [43, 49, 104]. Moreover, knockdown of PHB1 enhanced ROS production [14, 94–96], whereas exogenous PHB1 not only decreased basal intracellular ROS levels [105] but also decreased ROS production and prevented apoptosis in stress conditions [57, 96, 105–108], suggesting that PHB1 is an endogenous protective protein that can alleviate damage due to oxidative stress. Because ETC is the major source of ROS generation and PHB1 directly regulates its activity [93, 97], it is not surprising that PHB1 is in control of ROS production. It seems that PHB1 dampens complex I-generated ROS, but not complex-III [96], which is also the site of superoxide production. In addition to suppressing ROS production, PHB1 also upregulates antioxidant enzymes to combat excess ROS. A handful of experimental observations depict how PHB1 is involved in the maintenance of ROS homeostasis. PHB1 overexpression restores the GSH/glutathione S-transferase antioxidant system [109] and activates antioxidant response element (ARE) 4 as well as the transcription of the antioxidant enzymes NAD(P)H quinone oxidoreductase-1 (NQO-1) and heme oxygenase-1 (HO-1), ultimately reducing the incidence of apoptosis [105]. Further investigation suggests that the activation of ARE is due to the activation of ERK, which has been shown to contribute to ARE activation through activation of the transcription factor AP-1 [105]. In PHB1-overexpression colitis mice models, ERK1/2, c-Fos, c-Jun, HO-1, and NQO-1 levels all increased [105]. Although nuclear factor erythroid 2-related factor 2 (Nrf2) is also the principal transcription factor that binds to ARE [110] and Nrf2 mRNA, and Nrf2 protein expression as well as Nrf2/DNA binding is sustained in PHB1 overexpression under oxidative stress [106], the upregulation of antioxidants HO-1 and NQO-1 occurs via a mechanism independent of Nrf2 in this condition [105].

In addition to the influence on mitochondrial dynamics, mitochondrial morphology, mitochondrial genome, ETC, and ROS, mitochondrial PHB complex also interacts with the anti-apoptotic Hax-1 protein. Hax-1, the hematopoietic cell-specific protein-associated protein X-1, is a nucleocytoplasmic shuttling protein and functions in the apoptotic cascade upstream from caspase activation [2]. In Hela cells, PHB2 directly bound to Hax-1 in the mitochondria [2]. And knockdown of PHB2 reduced Hax-1 expression and induced caspase-dependent apoptosis [2]. In the mitochondria, Hax-1 is integrated in the outer mitochondrial membrane and localized in the intermembrane space. Because Hax-1 can be proteolytically degraded by the Omi/HtrA2 protease [111], it is suspected to be protected by the PHB complex. It is worth noting that knockdown of Hax-1 induces apoptotic cell death without damaging normal mitochondrial morphology [2].

Overall, deficiency in the PHB complex in the mitochondria may lead to programmed cell death due to its adverse impact on mitochondrial dynamics, mitochondrial morphology, mitochondrial genome, ETC, ROS homeostasis, as well as other anti-apoptotic proteins. Consequently, these events sustain and interact with each other, giving rise to a vicious cycle of mitochondrial dysfunction, furthering mitochondria-mediated apoptosis (Fig. 1).

## PHB post-transcriptional modification and the implication in apoptosis

Many mitochondrially localized proteins involved in the apoptotic process are functionally affected by phosphorylation, which can ultimately dictate whether a cell lives or dies [112]. PHB is one example. Studies have found that the phosphorylation sites of PHB1 include Ser^252^, Ser^254^, Sr^265^, and Thr^258^ as well as four tyrosine residues (Tyr^28^, Tyr^114^, Tyr^249^, and Tyr^259^) [113, 114]. The posttranslational modification of PHB potentiates the signal pathways involved in cell apoptosis, survival, and differentiation [113, 115–117]. Upon activation of primary human T cells, PHB1 was phosphorylated on serine residue(s), and PHB2 was phosphorylated on serine and tyrosine residues. These up-regulated proteins function to regulate mitochondrial homeostasis and T cell viability and differentiation [115]. In staurosporine-treated undifferentiated granulosa cells (GCs), increased mitochondrial PHB1 levels suppress the expression of the major pro-apoptotic factors Bax and Bak. MEK inhibitors abate the phosphorylation of PHB1 and enhance the release of cytochrome C and apoptosis [59]. Studies have also found that PHB is a substrate for Akt both in vitro and in vivo [30, 40, 117, 118]. In prostate cancer cells, TGF-β activation of the Raf-MEK-ERK-PKC-δ intracellular signal pathway leads to PHB1 phosphorylation and decreases inner mitochondrial permeability, cell survival, and cell invasion. In contrast, upregulating the 14-3-3 protein results in PKC-δ inhibition, PHB1 dephosphorylation, and increased cell apoptosis [40]. The phosphorylation status of PHB1 may act as a “molecular switch” for cell apoptosis during TGF-β stimulation. In MiaPaCa-2 pancreatic cancer cells, Akt phosphorylates PHB1 at Thr^258^ but does not phosphorylate PHB2 [117]. In NB4 human leukemia cells, PHB2 is phosphorylated by Akt on Ser^91^ and Ser^176^, and dephosphorylation of PHB2 Ser^91^ results in rapid cellular apoptosis [118]. The phosphorylation status of PHB1 can also affects the phosphorylation or activation of Akt [33, 57, 58, 68, 95, 119]. For example, phosphorylation of PHB1 on Thr^258^ in the raft domain of the plasma membrane is required for the activation of Ras-induced Raf-MEK-ERK and the PI3 K/Akt pathways, which promote tumor cell proliferation and metastasis [33, 119]. This indicate a complicated regulation network between the prosurvival kinase Akt and its substrate PHB. Apart from phosphorylation, PHB is also regulated by several other post-transcriptional modifications, such as O-GlcNAc modifications, palmitoylation, transamidation, and tyrosine nitrosylation [114]. Because O-GlcNAc modification and tyrosine phosphorylation of PHB can affect each other [116], whether there is any association between apoptosis regulation and other post-transcriptional modifications warrants further investigation. The delineation of the regulation and functional consequences of these post-transcriptional events will be important to understanding the regulatory complexity of apoptosis.

Another potential mechanism of post-transcriptional regulation of PHB is mediated by microRNA. MicroRNA can directly bind to the PHB 3′-UTR and induce cleavage of PHB mRNA, resulting in loss of function. In glioma cells, the oncomiR microRNA-26a promotes tumor growth and angiogenesis by targeting PHB1 [120]. Similarly, in prostate cancer and gastric cancer cells, microRNA-27a promotes tumor growth by directly targeting PHB1 [121]. However, in glioma cells, microRNA-27a promotes apoptosis by decreasing PHB1 protein expression [122]. Likewise, in mature gonadotropes, microRNA-27 downregulates PHB1 protein levels, and this is associated with an apoptotic response in the normal development and function of the reproductive axis [8]. In cardiomyocytes, miR-539 targets PHB2 and induces mitochondrial fission and apoptosis under anoxia injury. Cardiac apoptosis-related lncRNA (CARL) inhibits cell apoptosis by impairing miR-539-dependent PHB2 downregulation [67]. Therefore, not only PHB proteins levels but also its activity may ultimately determine cell fate.

## Conclusions

Taken together, the data presented here point to a critical role of PHB in cell survival and apoptosis. Importantly, the contribution of PHB to apoptosis is dependent on their cellular localization, the cell type, and post-transcriptional modifications. Because altered PHB signaling is implicated in a range of apoptosis-associated events, intense efforts should be directed at improving the understanding of the nuclear trafficking and transcriptional regulation of PHB and its target genes. Targeting PHB may have inspiring prospects in the future therapy of apoptosis-associated diseases.
